# Supplementary material for: Cyclophilin A supports translation of intrinsically disordered proteins and affects haematopoietic stem cell ageing
Source: Nat Cell Biol. 2024 Mar 29;26(4):593–603. doi: 10.1038/s41556-024-01387-x (PMC11021199; doi:10.1038/s41556-024-01387-x)
Supplement: Supplementary file 1 — Supplementary Figs. 1–32, Tables 1–11 and notes 1–5. [file 41556_2024_1387_MOESM1_ESM.pdf]

nature cell biology

Article

https://doi.org/10.1038/s41556-024-01387-x

Cyclophilin A supports translation of intrinsically disordered proteins and affects haematopoietic stem cell ageing

In the format provided by the authors and unedited

SI-Video legends: Impaired stress granule formation in absence of PPIA. HeLa cells were transfected with PABPC1-GFP and condensation was observed for 35 minutes, with frames taken every 30 seconds, following treatment with sodium arsenite at 1:200 dilution under tissue culture conditions:

Video 1: PABP-GFP phase separation in wild-type HeLa cells;

Video 2: Stress granule formation in negative control knockdown HeLa cells;

Video 3: Phase separation is impaired in *PPIA* knockdown HeLa cells (Kd1).
