# Supplementary material for: Cyclophilin A supports translation of intrinsically disordered proteins and affects haematopoietic stem cell ageing
Source: Nat Cell Biol. 2024 Mar 29;26(4):593–603. doi: 10.1038/s41556-024-01387-x (PMC11021199; doi:10.1038/s41556-024-01387-x)
Supplement: Supplementary file 2 — Reporting Summary [file 41556_2024_1387_MOESM2_ESM.pdf]

nature portfolio

Corresponding author(s): Andre Catic

Last updated by author(s): Feb 2, 2024

Reporting Summary

Nature Portfolio wishes to improve the reproducibility of the work that we publish. This form provides structure for consistency and transparency in reporting. For further information on Nature Portfolio policies, see our Editorial Policies and the Editorial Policy Checklist.

Statistics

For all statistical analyses, confirm that the following items are present in the figure legend, table legend, main text, or Methods section.

n/a	Confirmed	
☐	☒	The exact sample size (*n*) for each experimental group/condition, given as a discrete number and unit of measurement
☐	☒	A statement on whether measurements were taken from distinct samples or whether the same sample was measured repeatedly
☐	☒	The statistical test(s) used AND whether they are one- or two-sided<br>*Only common tests should be described solely by name; describe more complex techniques in the Methods section.*
☒	☐	A description of all covariates tested
☒	☐	A description of any assumptions or corrections, such as tests of normality and adjustment for multiple comparisons
☐	☒	A full description of the statistical parameters including central tendency (e.g. means) or other basic estimates (e.g. regression coefficient) AND variation (e.g. standard deviation) or associated estimates of uncertainty (e.g. confidence intervals)
☐	☒	For null hypothesis testing, the test statistic (e.g. *F*, *t*, *r*) with confidence intervals, effect sizes, degrees of freedom and *P* value noted<br>*Give P values as exact values whenever suitable.*
☒	☐	For Bayesian analysis, information on the choice of priors and Markov chain Monte Carlo settings
☒	☐	For hierarchical and complex designs, identification of the appropriate level for tests and full reporting of outcomes
☒	☐	Estimates of effect sizes (e.g. Cohen's *d*, Pearson's *r*), indicating how they were calculated

Our web collection on statistics for biologists contains articles on many of the points above.

Software and code

Policy information about availability of computer code

Data collection	BD FACS DiVa software 9.0 was used to acquire flow cytometry data. Commercial softwares used to collect MS/MS data, RNA sequencing data, and capture microscopy pictures are indicated in the Methods section of the manuscript. No custom algorithm or custom software was used.
Data analysis	All open source and commercial software and proteomic databases used to analyse MS/MS data, RNA sequencing data and microscopy pictures are described in the Methods section of the manuscript. All statistical analyses were performed using Stata v.15.1 and GraphPad Prism 10. Image analysis was performed with FIJI/ImageJ 2.00/1.52p and ZenPro 3.1. 2D gel captures were analysed with DeCyder 7.0 and ImageQuant(GE Healthcare). Pulsed SILAC data was analysed with Qlucore Omics Explorer 3.5 software. FACS data was analysed with FlowJo version 10. Gene Set Enrichment Analyses were performed with version 4.3.2. The 3-D molecular structure of PPIA protein was visualized with PyMOL v.2.5.2 (licensed by A.C.). Rendered graphics were created with BioRender (licensed by L.M.).

For manuscripts utilizing custom algorithms or software that are central to the research but not yet described in published literature, software must be made available to editors and reviewers. We strongly encourage code deposition in a community repository (e.g. GitHub). See the Nature Portfolio guidelines for submitting code & software for further information.

Data

Policy information about availability of data

All manuscripts must include a data availability statement. This statement should provide the following information, where applicable:

- Accession codes, unique identifiers, or web links for publicly available datasets
- A description of any restrictions on data availability
- For clinical datasets or third party data, please ensure that the statement adheres to our policy

All data necessary for interpreting, verifying, and extending the research in this article have been co-submitted as source data and supplementary information files. Raw data have been deposited in the repositories outlined below and are available without restrictions.

Mass spectrometry data obtained after 3XF-PPIA immunoprecipitation (Fig. 3c) are deposited with the ProteomeXchange Consortium via the MassIVE repository (MSV000083867) with the dataset identifier PXD014025 (https://massive.ucsd.edu/). The datasets generated in the mouse HSC proteome profiling (Extended Data Fig. 4b) have been deposited to the ProteomeXchange Consortium via the MassIVE repository (MSV000083845) with the dataset identifier PXD013995. Proteome data following isobaric labelling and pulsed SILAC is available as supplementary information and at MassIVE (MSV000093125, MSV000093126, MSV000093127) and ProteomeXchange (PXD046245, PXD046246, PXD046247).

For transcriptomic analysis of murine HSPCs (SI-Mouse HSPCs RNA seq), raw and processed RNA-seq data have been deposited with the Gene Expression Omnibus (GEO) database under accession code GSE151125.

Previously published proteome data that were re-analysed are available under accession codes PXD007048 (proteome data) and GSE115353 (transcriptomics).

The PPIA structure was previously submitted to the Research Collaboratory for Structural Bioinformatics – Protein Data Bank under accession 7ABT.

PhaSepDB2.0 contains a database of 2,957 non-redundant phase-separation proteins and membrane-less organelles (http://db.phasep.pro/), curated from literature and databases.

IUPred2A was used to compute the likelihood of structural disorder per residue (https://iupred2a.elte.hu/).

Research involving human participants, their data, or biological material

Policy information about studies with human participants or human data. See also policy information about sex, gender (identity/presentation), and sexual orientation and race, ethnicity and racism.

Reporting on sex and gender	N/A
Reporting on race, ethnicity, or other socially relevant groupings	N/A
Population characteristics	N/A
Recruitment	N/A
Ethics oversight	N/A

Note that full information on the approval of the study protocol must also be provided in the manuscript.

Field-specific reporting

Please select the one below that is the best fit for your research. If you are not sure, read the appropriate sections before making your selection.

☒ Life sciences ☐ Behavioural & social sciences ☐ Ecological, evolutionary & environmental sciences

For a reference copy of the document with all sections, see nature.com/documents/nr-reporting-summary-flat.pdf

Life sciences study design

All studies must disclose on these points even when the disclosure is negative.

Sample size	A priori power calculations were conducted to ascertain the required sample size for each of two equal-sized groups. The effect size was established at 15% of the pooled standard deviations, estimated at 10% based on historical data from our group. This analysis was predicated on performing a two-tailed independent samples test, appropriate for testing without a predetermined direction. Power was validated using post hoc verification.
Data exclusions	No data were excluded from this study. Animals with signs of sickness were euthanized.
Replication	For transplantation studies involving Ppia heterozygous versus knockout donor cells, two separate experiments were conducted, one with

Replication	male and one with female donor mice, showing identical results. Other experiments that were performed in duplicates or triplicates are described in the Methods section and Figure Legends.
Randomization	Randomization was not feasible within the experimental design. However, in transplantation studies, transplant recipient animals were randomly assigned at the time of irradiation and donor cells were pooled from two or three animals. Experiments were age and gender matched and independently conducted with male/female mice.
Blinding	For transplantation studies, animal handling, microscopy, and FACS, experiments and analysis were handled by separate researchers. The FACS technician performing population statistics was blinded to the genotype of the donor mice.<br>For all imaging analyses, the experimenter was blinded to the origin of the samples.<br>For pulse SILAC and MS/MS, the researcher operating the mass spectrometrer and generating the raw proteomic data was blinded to the origin of the samples.

Reporting for specific materials, systems and methods

We require information from authors about some types of materials, experimental systems and methods used in many studies. Here, indicate whether each material, system or method listed is relevant to your study. If you are not sure if a list item applies to your research, read the appropriate section before selecting a response.

Materials & experimental systems

n/a	Involved in the study	
☐	☒	Antibodies
☐	☒	Eukaryotic cell lines
☒	☐	Palaeontology and archaeology
☐	☒	Animals and other organisms
☒	☐	Clinical data
☒	☐	Dual use research of concern
☒	☐	Plants

Methods

n/a	Involved in the study	
☒	☐	ChIP-seq
☐	☒	Flow cytometry
☒	☐	MRI-based neuroimaging

Antibodies

Antibodies used

Antigen & Fluorophore - (Vendor & Catalog #) - Dilution:

FACS - Stem and progenitor cells:

CD45.1-PE (Biolegend 110708) 1:200
CD45.1-APC (eBioscience 17-0453-82) 1:200
CD45.2-PE Cy5.5 (eBioscience 35-0454-82) 1:200
CD45.2-Pac Blue (Biolegend 109820) 1:200
cKit-APC (BD Biosciences 553356) 1:200
cKit-APC Cy7 (eBioscience 47-1171-82) 1:50
SA-Pac Orange (Invitrogen S32365) 1:200
SA-Pac Blue (Invitrogen S11222) 1:200
Sca1-Pac Blue (Biolegend 108120) 1:200
Sca1-PE Cy5.5 (Invitrogen MSCA18) 1:300
Sca1-PE Cy7 (Biolegend 108114) 1:200
CD34-FITC (eBioscience 11-0341-85) 1:50
CD127-APC Cy7 (eBioscience 47-1271-82) 1:200
CD135-PE BD (Biosciences 553842) 1:50
CD150-PE Cy7 (Biolegend 115914) 1:200
CD16/32-PE-Cy7 (eBioscience 25-0161-82) 1:200

FACS - Peripheral blood:

CD45.1-PE (Biolegend 110708) 1:125
CD45.2-BV421 (Biolegend 109832) 1:125
CD3-PE Cy7 (eBioscience 25-0031-81) 1:125
B220-PerCP Cy5.5 (eBioscience 45-0452-80) 1:125
MAC1-APC (eBioscience 17-0112-81) 1:125
GR1-APC eF780 (eBioscience 47-5931-80) 1:125

Western and IP antibodies:

PPIA (CST) 2175 1:1,000
PPIA (Abcam) ab1791 1:1,000
PPIA (Abcam) ab58144 IP
FLAG (Sigma) F1804 1:1,000
IgG1 control (CST) 5415 1:1,000
HA (Sigma) 11867423001 1:1,000

H3 (Abcam) ab1791 1:1,000
PABPC1 (CST) 4992 1:1,000
Caprin1 (Proteintech) 15112-1-AP 1:1,000
DDX6 (Proteintech) 14632-1-AP 1:1,000
NPM1 (Proteintech) 10306-1-AP 1:5,000
G3BP1 (Proteintech) 13057-2-AP 1:1,000
beta-Tubulin (CST) 86298 1:3,000
GAPDH (Abcam) ab204481 1:10,000

Proximity Ligation Assay:

PPIA (Abcam) ab58114 1:100
PPIA (Abcam) ab41684 1:100
PABPC1 (Proteintech) 10970-1-AP 1:100
DDX6 (Proteintech) 14632-1-AP 1:100
NPM1 (Proteintech) 10306-1-AP 1:100

Immuno-Fluorescence:

G3BP1 (Proteintech) 13057-2-AP 1:500
DDX6 (Proteintech) 14632-1-AP 1:100

Validation

All antibodies were commercially purchased and validated for their respective application by the manufacturer.

For flow cytometry:
B220-PerCP Cy5.5 (eBioscience/Thermo Scientific, Cat. #45-0452-80, Clone RA3-6B2). This RA3-6B2 antibody has been tested by flow cytometric analysis of mouse splenocytes. Advanced Verification: this antibody was verified by relative expression to ensure that the antibody binds to the antigen stated.

c-Kit (CD117)-APC (BD Biosciences, Cat. #553356, Clone 2B8). Application: Flow cytometry (Routinely rested). A single-cell suspension of BALB/c bone marrow was simultaneously stained with FITC Rat Anti-Mouse CD45R/B220 (Cat. Nos. 553087/553088, both panels) and either APC Rat IgG2b, κ Isotype Control (Cat. No. 553991) or APC Rat Anti-Mouse CD117 (Cat. No. 553356) monoclonal antibodies. Flow cytometry was performed on a BD FACSCalibur flow cytometry system.

c-Kit (CD117)-APC-Cy7 (eBioscience/Thermo Scientific, Cat. #47-1171-82, Clone 2B8). Applications Tested: this 2B8 antibody has been tested by flow cytometric analysis of mouse bone marrow cells.

Gr-1 (Ly-6G/Ly-6C)-APC eF780 (eBioscience/Thermo Scientific, Cat. #47-931-80, Clone RB6-8C5). This RB6-8C5 antibody has been tested by flow cytometric analysis of mouse bone marrow cells. Advanced verification: this Antibody was verified by relative expression to ensure that the antibody binds to the antigen stated.

MAC1 (CD11b)-APC (eBioscience/Thermo Scientific, Cat. #17-0112-81, Clone M1/70). The M1/70 antibody has been tested by flow cytometric analysis of mouse splenocytes.

Sca1-Pac Blue (BioLegend, Cat. #108120, Clone D7). Each lot of this antibody is quality control tested by immunofluorescent staining with flow cytometric analysis.

Sca1-PE Cy5.5 (Invitrogen/Thermo Scientific, Cat. #MSCA18, Clone D7). The antibody has been discontinued and validation information is no longer available on the website.

Sca1-PE Cy7 (BioLegend, Cat. #108114, Clone D7). Each lot of this antibody is quality control tested by immunofluorescent staining with flow cytometric analysis.

Streptavidin-Pac Orange (Invitrogen/Thermo Scientific, Cat. #S32365). For Use With (Application): Flow Cytometry, Immunoassays, Histochemical Applications, Blot Analysis. Not an antibody, no precise validation data is available. Certificate of Analysis only.

Streptavidin-Pac Blue (Invitrogen/Thermo Scientific, Cat. #S11222). For Use With (Equipment): Flow Cytometry, Immunoassays, Histochemical Applications, Blot Analysis. Not an antibody, no precise validation data is available. Certificate of Analysis only.

CD3-PE Cy7 (eBioscience/Thermo Scientific, Cat. #25-0031-81, Clone 145-2C11). This 145-2C11 antibody has been tested by flow cytometric analysis of mouse thymocytes and splenocytes.

CD16/32-PE Cy7 (eBioscience/Thermo Scientific, Cat. #25-0161-82, Clone 93). This 93 antibody has been tested by flow cytometric analysis of mouse splenocytes.

CD34-FITC (eBioscience/Thermo Scientific, Cat. #11-0341-85, Clone RAM34). This RAM34 antibody has been tested by flow cytometric analysis of mouse bone marrow cells.

CD45.1-PE (BioLegend, Cat. # 110708, Clone A20). Each lot of this antibody is quality control tested by immunofluorescent staining with flow cytometric analysis.

CD45.1-APC (eBioscience/Thermo Scientific, Cat. # 17-043-82, Clone A20). Applications Tested: the A20 antibody has been tested by flow cytometric analysis of mouse splenocytes.

CD45.2-PE Cy5.5 (eBioscience/Thermo Scientific, Cat. #35-0454-82, Clone 104). Applications Tested: this 104 antibody has been

tested by flow cytometric analysis of BALB/c splenocytes.

CD45.2-Pac Blue (BioLegend, Cat. #109820, Clone 104). Each lot of this antibody is quality control tested by immunofluorescent staining with flow cytometric analysis.

CD45.2-BV421 (BioLegend, Cat. #109832, Clone 104). Each lot of this antibody is quality control tested by immunofluorescent staining with flow cytometric analysis.

CD127-APC Cy7 (eBioscience/Thermo Scientific, Cat. #47-1271-82, Clone A7R34). This A7R34 antibody has been tested by flow cytometric analysis of mouse splenocytes.

CD135-PE (BD Biosciences, Cat. #553842, Clone A2F10.1). Application: Flow cytometry (Routinely Tested). In flow cytometric analysis, the A2F10 antibody recognizes Flt3-transfected Y3 cells (rat myeloma), but not the parent cell line in addition to recognizing early B lymphoid lineage cells in juvenile and adult bone marrow.

CD150-PE Cy7 (BioLegend, Cat. #115914, Clone TC15-12F12.2). Each lot of this antibody is quality control tested by immunofluorescent staining with flow cytometric analysis.

For Western blots and Immunoprecipitation assays:
PPIA (Cell Signaling Technology, Cat. #2175). Cyclophilin A Antibody detects endogenous levels of total Cyclophilin A protein. Application: Western Blotting, Dilution 1:1000. Species reactivity is determined by testing in at least one approved application (e.g., western blot).

PPIA (Abcam, Cat. # ab41684). Replenishment batches of our polyclonal antibody, ab41684 are tested in WB. Previous batches were additionally validated in ICC/IF.

PPIA (Abcam, Cat. #58144, Clone 1F4-1B5). Our Abpromise guarantee covers the use of ab58144 in the following tested applications: WB, ICC/IF, IP, flow cytometry. Western blot: Use at an assay dependent concentration. Predicted molecular weight: 18 kDa.

FLAG (Millipore Sigma, Cat. #F1804, Clone M2). Application: for highly sensitive and specific detection of FLAG fusion proteins by immunoblotting, immunoprecipitation, immunohistochemisty, immunofluorescence and immunocytochemistry. Optimized for single banded detection of FLAG fusion proteins in mammalian, plant, and bacterial expression systems. Specificity: Conforms. Detects a single band of protein on a Western Blot from mammalian crude cell lysates. Sensitivity test: Conforms. Detects 2 ng of FLAG-BAP fusion protein by Dot Blot using Chemiluminescent Detection.

IgG1 Isotype Control (Cell Signaling Technology, Cat. #5415). Mouse (G3A1) mAb IgG1 Isotype Control is not directed against any known antigen. It functions as an isotype control for mouse IgG1 monoclonal antibodies. Species reactivity is determined by testing in at least one approved application (e.g., western blot).

HA tag (Sigma-Aldrich, Cat. #11867423001, Clone 3F10). Quality: function tested in western blot. Use Anti-HA High Affinity for the detection of native influenza hemagglutinin protein and recombinant proteins that contain the HA epitope using Dot blots, ELISA, Immunocytochemistry, Immunoprecipitation, and Western blots.

Histone H3 (Abcam, Cat. #1791). WB: Detects a band of approximately 17 kDa (predicted molecular weight: 15 kDa). Specificity: based only on sequence homology, we expect the antibody to react with multiple variants of H3 such as H3.1, H3.2 and H3.3.

PABPC1 (Cell Signaling Technology, Cat. #4992). PABP1 Antibody detects endogenous levels of total PABP1 and PABP3 proteins. Species reactivity is determined by testing in at least one approved application (e.g., western blot).

Caprin1 (Proteintech, Cat. #15112-1-AP). Positive WB detected in HEK-293 cells, mouse brain tissue, HEK-293T cells, rat brain tissue, HeLa cells, Jurkat cells, NIH/3T3 cells.

DDX6 (Proteintech, Cat. #14632-1-AP). 14632-1-AP targets DDX6 in WB, IP, IHC, IF, ELISA applications and shows reactivity with human, mouse, rat samples. Positive WB detected in HeLa cells, HEK-293 cells, HepG2 cells, Jurkat cells, K-562 cells, C2C12 cells, mouse testis tissue.

NPM1 (Proteintech, Cat. #10306-1-AP). 10306-1-AP targets B23/NPM1 in WB, IP, IHC, IF, CoIP, ChIP, ELISA applications and shows reactivity with human, rat samples. Positive WB detected in COLO 320 cells, Jurkat cells, multi-cells, K-562 cells, HeLa cells, HEK-293 cells.

G3BP1 (Proteintech, Cat. #13057-2-AP). 13057-2-AP targets G3BP1 in WB, RIP, IP, IHC, IF, FC, CoIP, ELISA applications and shows reactivity with human, rat, mouse samples. Positive WB detected in C6 cells, HEK-293 cells, human brain tissue, Neuro-2a cells, HeLa cells, HepG2 cells, MCF-7 cells, Jurkat cells, mouse kidney tissue, rat kidney tissue, mouse brain tissue, rat brain tissue. The protein is known to run at higher than expected molecular weight in SDS PAGE.

Beta-Tubulin (Cell Signaling Technology, Cat. #86298, Clone D3U1W). β-Tubulin (D3U1W) Mouse mAb recognizes endogenous levels of total β-tubulin protein. Species reactivity is determined by testing in at least one approved application (e.g., western blot).

GAPDH (Abcam, Cat. #ab204481, Clone EPR16884). WB: detects a band of approximately 36 kDa (predicted molecular weight: 36 kDa). Species reactivity: reacts with: Mouse, Rat, Human.

For Proximity Ligation Assays:
PPIA (Abcam, Cat. # ab41684). Replenishment batches of our polyclonal antibody, ab41684 are tested in WB. Previous batches were additionally validated in ICC/IF.

PPIA (Abcam, Cat. #58144, Clone 1F4-1B5). Our Abpromise guarantee covers the use of ab58144 in the following tested applications:

WB, ICC/IF, IP, flow cytometry. Western blot: Use at an assay dependent concentration. Predicted molecular weight: 18 kDa.

PABPC1 (Proteintech, Cat. #10970-1-AP). 10970-1-AP targets PABPC1, PABP in WB, IP, IHC, IF, FC, ELISA applications and shows reactivity with human, mouse, rat samples. Positive IF detected in MCF-7 cells.

DDX6 (Proteintech, Cat. #14632-1-AP). 14632-1-AP targets DDX6 in WB, IP, IHC, IF, ELISA applications and shows reactivity with human, mouse, rat samples. Positive IF detected in HeLa cells, hTERT-RPE1 cells.

NPM1 (Proteintech, Cat. #10306-1-AP). 10306-1-AP targets B23/NPM1 in WB, IP, IHC, IF, CoIP, chIP, ELISA applications and shows reactivity with human, rat samples. Positive IF detected in HeLa cells.

Immuno-fluorescence:
G3BP1 (Proteintech, Cat. #13057-2-AP). 13057-2-AP targets G3BP1 in WB, RIP, IP, IHC, IF, FC, CoIP, ELISA applications and shows reactivity with human, rat, mouse samples. Positive IF detected in sodium arsenite treated HeLa cells.

DDX6 (Proteintech, Cat. #14632-1-AP). 14632-1-AP targets DDX6 in WB, IP, IHC, IF, ELISA applications and shows reactivity with human, mouse, rat samples. Positive IF detected in HeLa cells, hTERT-RPE1 cells.

Eukaryotic cell lines

Policy information about cell lines and Sex and Gender in Research

Cell line source(s)

Cell lines were purchased from ATCC (293T CRL-3216; HeLa CCL-2) or DMSZ (NB4 ACC-207; OCI-AML3 ACC-582), cultured with the medium composition recommended by the supplier, and monitored for signs of infection, including mycoplasma contamination.

Authentication

The ATCC cell lines were confirmed by STR profiling and HPV positivity (HeLa).

Mycoplasma contamination

Cells were monitored for signs of infection, including mycoplasma contamination. Negative tests were recorded using the Lonza MycoAlert Mycoplasma Detection Kit.

Commonly misidentified lines
(See ICLAC register)

The study did not involve misidentified cell lines.

Animals and other research organisms

Policy information about studies involving animals; ARRIVE guidelines recommended for reporting animal research, and Sex and Gender in Research

Laboratory animals

We used C57BL/6 wild-type mice or derived Ppia-/- and Ppia+/- mice of both sexes, multiple ages as indicated in the Methods section. As recipients in transplant experiments, we used female C57BL/6.SJL mice from the Jackson Laboratory (catalot no. 002014). Animals were housed in groups of 5 or 4 (if weight >25g) in ventilated cages in a pathogen-free high-barrier facility under ambient temperature and humidity. The mice were on a standard rodent diet of chow and water ad libitum, under a 12-hour light/dark cycle.

Animal ages (details described in the Methods section):
Transplant donors and recipients: 3-6 months of age, except for Fig. 2 where the donors were 18 months of age.
Age-dependent transcriptome and proteome analyses: young animals are 3-6 months of age, aged animals are over 20 months of age.
Ppia genotype-dependent transcriptome and proteome analyses: mice are 10-12 months of age.
General proteome and PLA interaction assays: mice are 4-8 months of age.

Wild animals

The study did not involve wild animals.

Reporting on sex

Experiments were performed using single-sex donor cells. No differences were observed between male or female donors.

Field-collected samples

The study did not involve samples collected from the field.

Ethics oversight

All animal experiments and care procedures were conducted at the Massachusetts General Hospital or the Baylor College of Medicine facilities in accordance with the Institutional Animal Care and Use Committee (IACUC) protocols approved at each institution, in compliance with all relevant ethical regulations, and following guidelines from the National Institutes of Health Guide for the Care and Use of Laboratory Animals (approved protocol #AN6745). The animal facilities were approved by the Association for Assessment and Accreditation for Laboratory Animal Care International (AAALAC).

Note that full information on the approval of the study protocol must also be provided in the manuscript.

Flow Cytometry

Plots

Confirm that:

☒ The axis labels state the marker and fluorochrome used (e.g. CD4-FITC).

☒ The axis scales are clearly visible. Include numbers along axes only for bottom left plot of group (a 'group' is an analysis of identical markers).

☒ All plots are contour plots with outliers or pseudocolor plots.

☒ A numerical value for number of cells or percentage (with statistics) is provided.

Methodology

Sample preparation	Samples were prepared using standard protocols, with details outlined in the Methods section.
Instrument	BD Biosciences LSR Fortessa for analysis and Aria II for sorting.
Software	Analysis was performed using pre-installed BD Biosciences software FACS DiVa 9.0 and confirmed post-hoc with FlowJo v.10.
Cell population abundance	Relative and absolute cell numbers were calculated between various samples. While the relative numbers are shown in the manuscript, the absolute cell numbers showed consistent changes based on genotype of the donor animals.
Gating strategy	Standard gating strategy was performed, as detailed in the Methods section. Dead cells were excluded based on FSC/SSC scatter.

☒ Tick this box to confirm that a figure exemplifying the gating strategy is provided in the Supplementary Information.
