# Supplementary material for: Cyclophilin A supports translation of intrinsically disordered proteins and affects haematopoietic stem cell ageing
Source: Nat Cell Biol. 2024 Mar 29;26(4):593–603. doi: 10.1038/s41556-024-01387-x (PMC11021199; doi:10.1038/s41556-024-01387-x)

Uncropped Western Blot Images

Fig. 2c

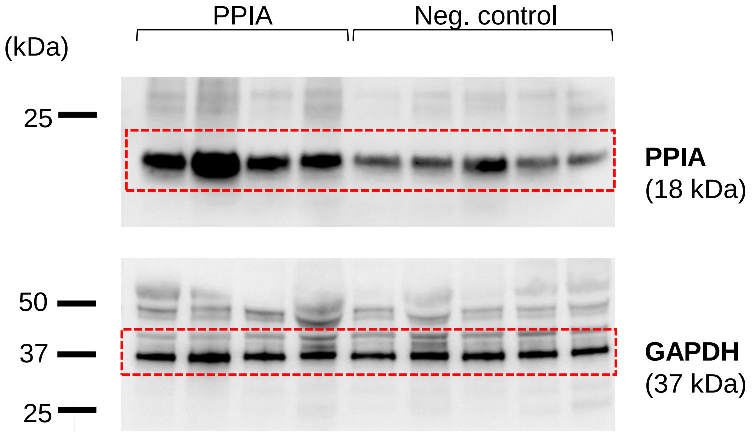

Fig. 4e, f

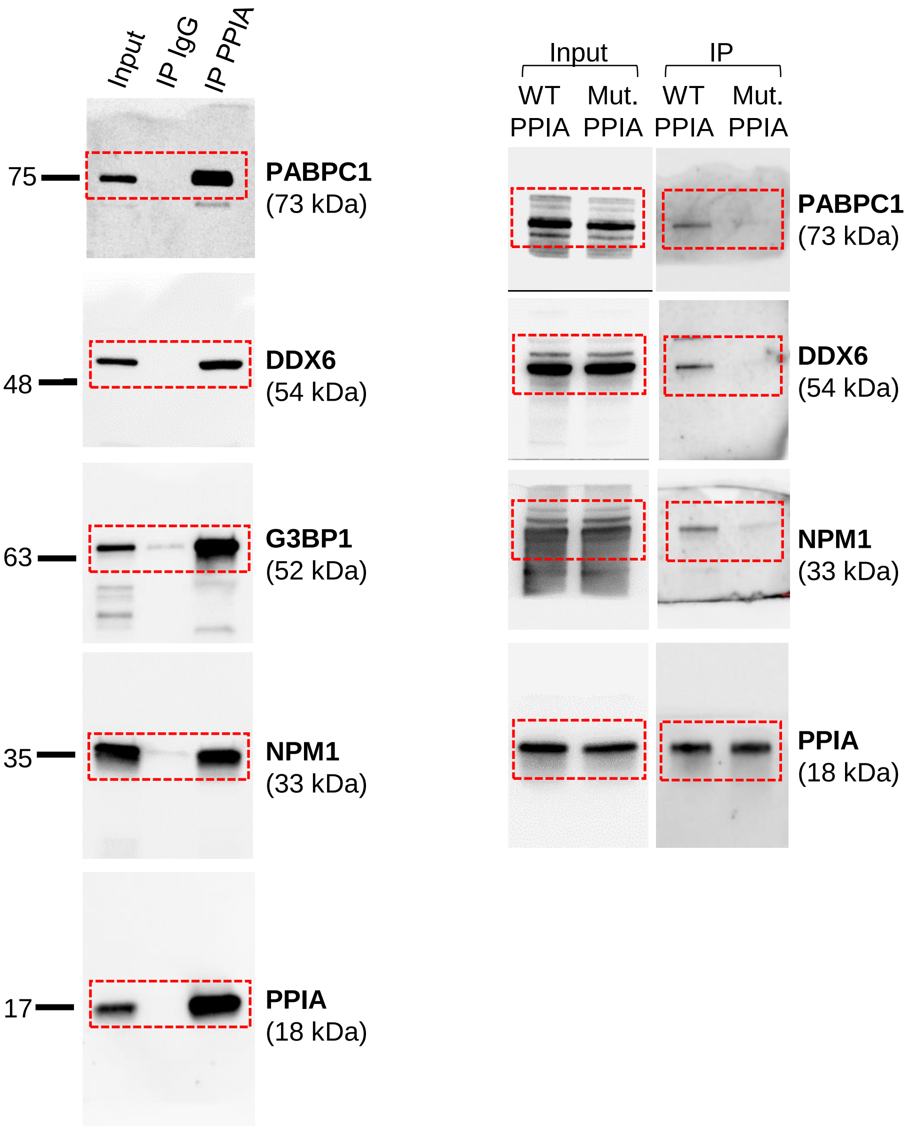

Fig. 4g

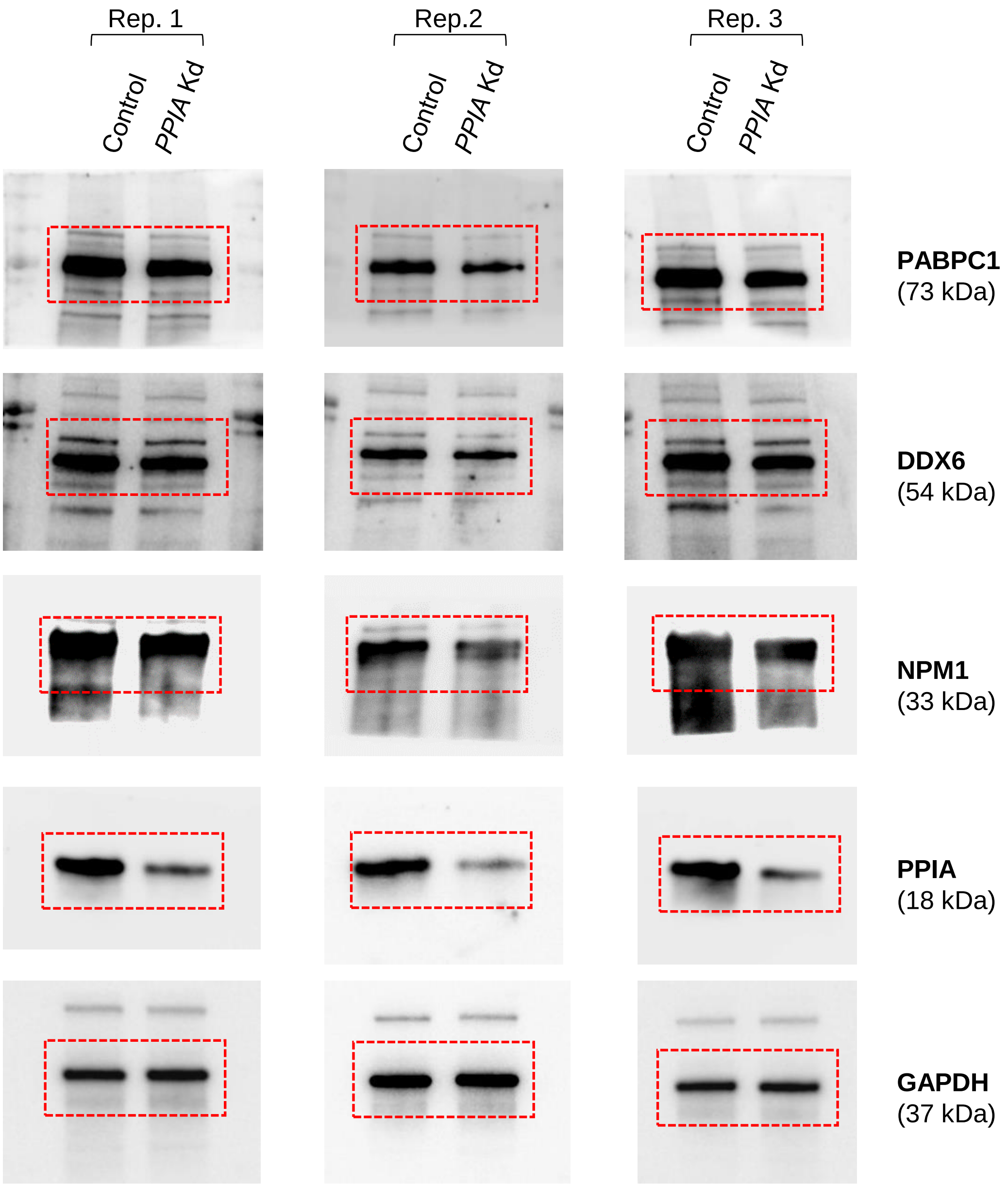
Rep. 1
Rep.2
Rep. 3
Control
PPIA Kd
PABPC1
(73 kDa)
DDX6
(54 kDa)
NPM1
(33 kDa)
PPIA
(18 kDa)
GAPDH
(37 kDa)

Extended Data Fig. 2d

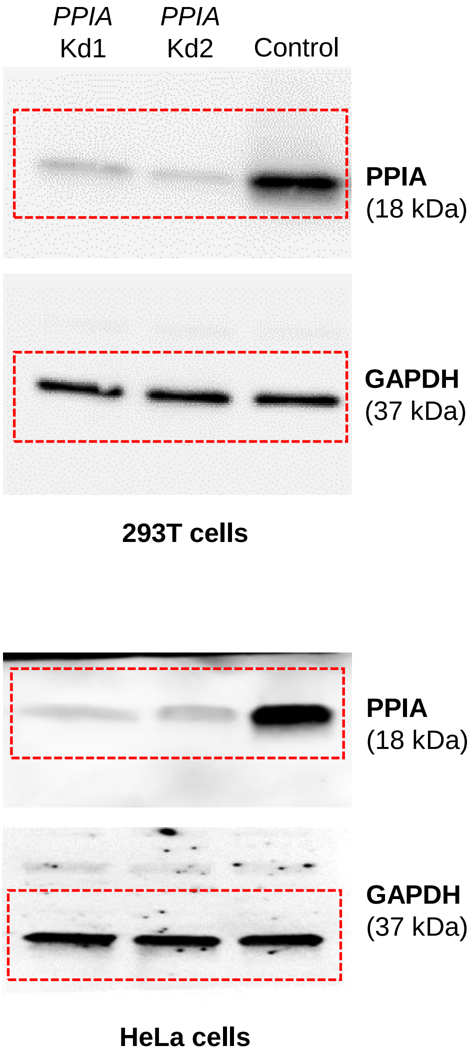

Extended Data Fig. 3b

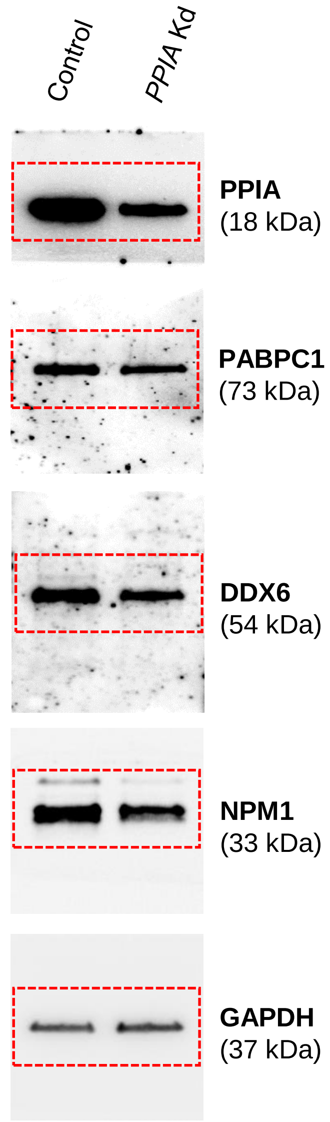

Extended Data Fig. 3c

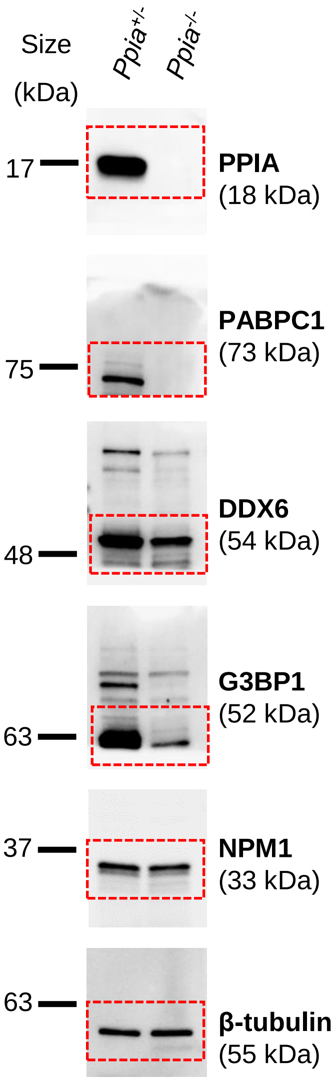

Extended Data Fig. 4e

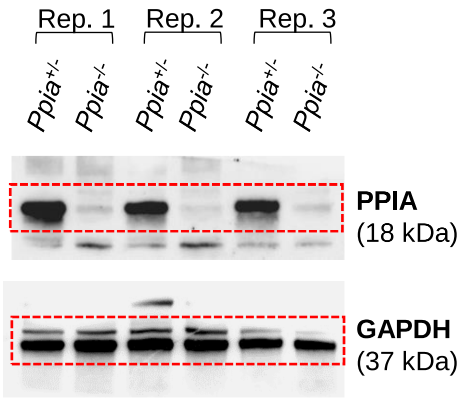

Supplement: Supplementary file 16 — Uncropped western blot images for all main and extended data figures. [file 41556_2024_1387_MOESM16_ESM.pdf]
