# Supplementary material for: Cyclophilin A supports translation of intrinsically disordered proteins and affects haematopoietic stem cell ageing
Source: Nat Cell Biol. 2024 Mar 29;26(4):593–603. doi: 10.1038/s41556-024-01387-x (PMC11021199; doi:10.1038/s41556-024-01387-x)

Supplementary Figure 1

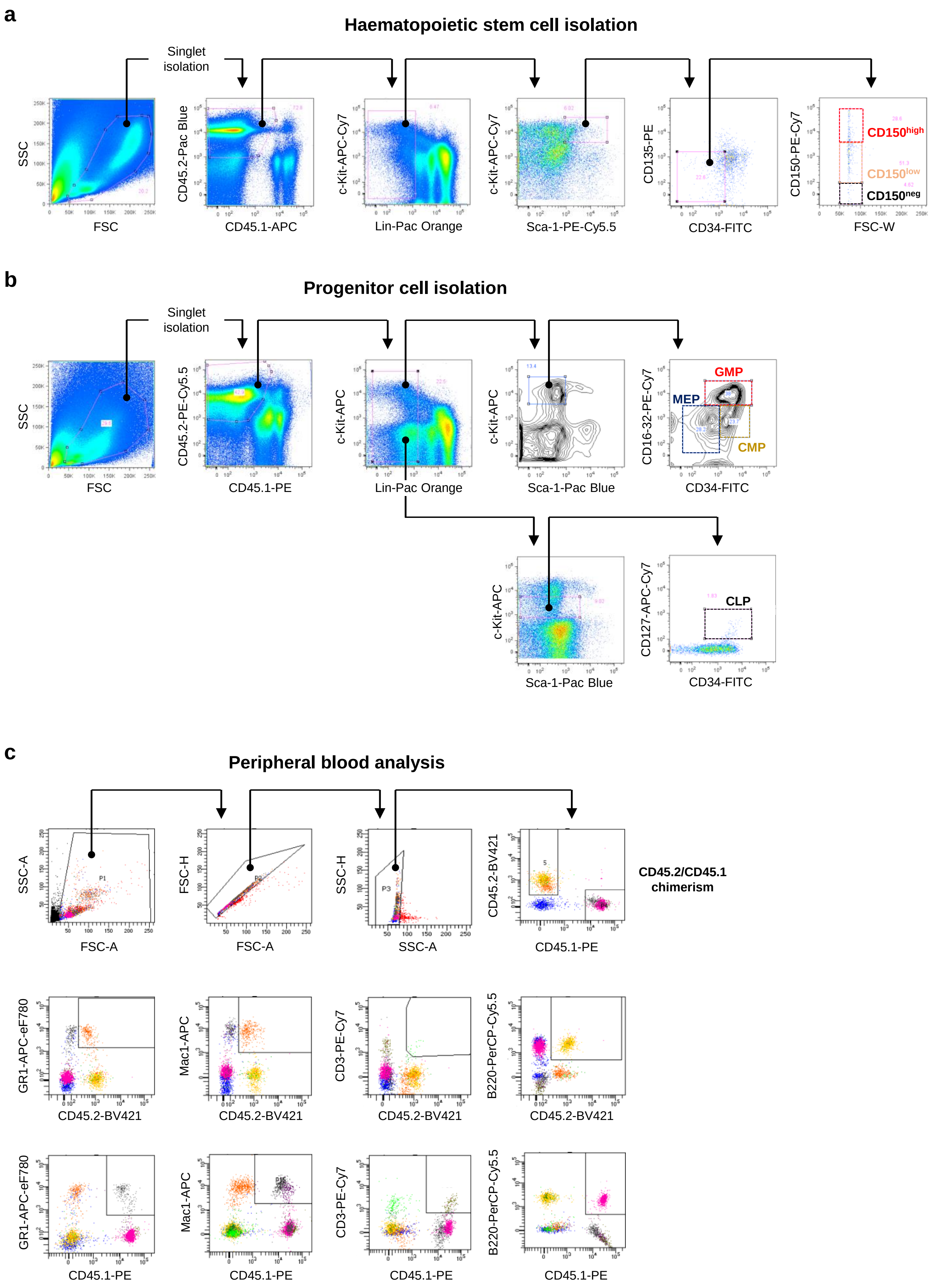

Supplement: Supplementary file 17 — Supplementary figure showing gating strategy. No legend provided. [file 41556_2024_1387_MOESM17_ESM.pdf]
